# 234. Risk Factors for Mortality in Patients with *Escherichia coli* Bloodstream Infections and Influence of the Antimicrobial Resistance Pattern in a Referral Hospital in Nicaragua

**DOI:** 10.1093/ofid/ofad500.307

**Published:** 2023-11-27

**Authors:** Mauricio Sánchez-Delgado, Sunaya Marenco-Avilés, Guillermo D Porras-Cortés

**Affiliations:** Hospital Dr. Fernando Vélez Paiz, Managua, Managua, Nicaragua; Hospital Dr. Fernando Vélez Paiz, Managua, Managua, Nicaragua; Hospital Dr. Fernando Vélez Paiz, Managua, Managua, Nicaragua

## Abstract

**Background:**

*Escherichia coli* is the most frequent etiological agent in bloodstream infections, and mortality has increased because of the increasing rates of bacterial resistance. Every healthcare institution has to characterize the epidemiological trend and recognize determinants for adverse outcomes in patients with bacteremia. The aim of this study was to identify risk factors for mortality and identify the influence of the pattern of bacterial resistance, as well as associated clinical characteristics in patients with *E. coli* bloodstream infections hospitalized at the Dr. Fernando Vélez Paiz Hospital in Managua, Nicaragua.

**Methods:**

It is a retrospective, cross-sectional, and analytical study. The study period was between January 1st, 2020, and December 31st, 2022. A total of 81 patients with bacteremia due to *E. coli* were analyzed out of the total number of patients with bloodstream infections (Figure 1). The epidemiological trend, clinical variables, and pattern of antimicrobial resistance were analyzed. Risk factors for mortality were identified, and odds ratios and confidence intervals were calculated.

**Figure 1.**
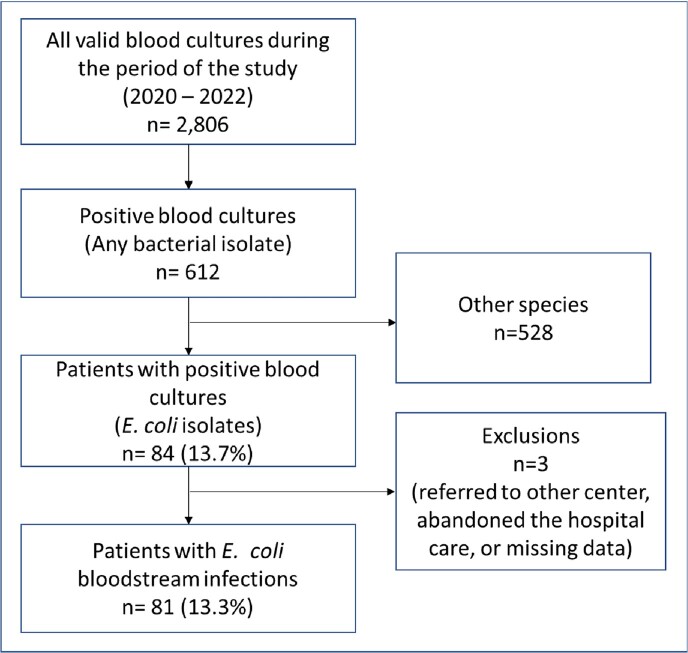
Flowchart of Selection of Patients with E. coli Bloodstream Infections

**Results:**

The prevalence of *E. coli* bacteremia among all bloodstream infections was 13.3%, with a mortality rate of 37.0%. The mean age of the patients was 54.7 ± 17.2 years old. Regarding comorbidities, 43.2% and 42.0% had hypertension and diabetes, respectively. Genitourinary was the main associated site of infection (58.0%) (Table 1). A high rate of *E. coli* producing ESBL was identified (64.2%). The resistance to ciprofloxacin and carbapenem was 67.9% and 12.3% respectively. Multi-drug resistance was observed in 28.4% of the strains. (Table 2). A history of steroid use was recorded in 40% of non-survivors versus 9.8% of survivors. Resistance to carbapenems was higher in non-survivors (26.7% vs. 3.9%). The main risk factors for mortality were use of a urinary catheter in ≥60 years old, resistance to carbapenems, and abdominal sepsis as source of infection, among others (Table 3).

**Table 1.**
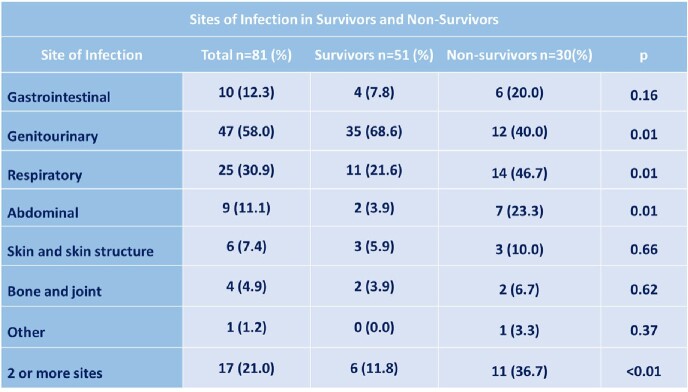
Sites of Infection in Survivors and Non-Survivors

**Table 2.**
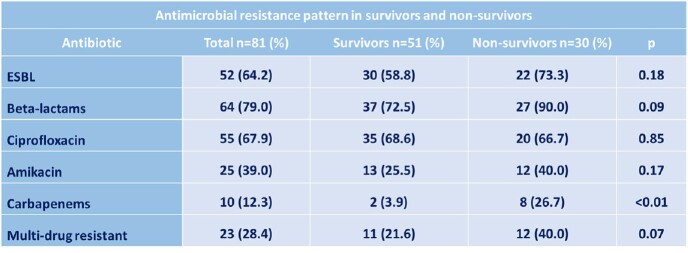
Antimicrobial Resistance Pattern in Survivors and Non-Survivors

**Table 3.**
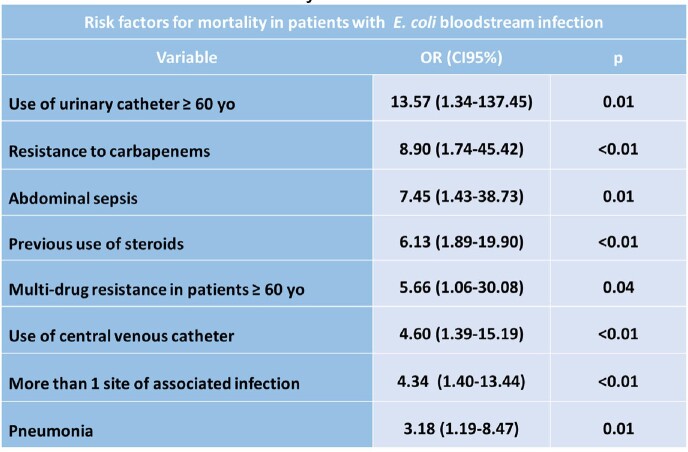
Risk Factor for Mortality in Patients with E. coli Bloodstream Infections

**Conclusion:**

The main risk factors for mortality in patients with bloodstream infections due to *E. coli* were use of urinary catheter in older patients, resistance to carbapenems, bacteremia associated with abdominal sepsis, among others. A high rate of resistance to ciprofloxacin, and beta-lactams was observed.

**Disclosures:**

**All Authors**: No reported disclosures

